# IP-10 measured by Dry Plasma Spots as biomarker for therapy responses in *Mycobacterium Tuberculosis* infection

**DOI:** 10.1038/srep09223

**Published:** 2015-03-18

**Authors:** Kristian Tonby, Morten Ruhwald, Dag Kvale, Anne Ma Dyrhol-Riise

**Affiliations:** 1 Institute of Clinical Medicine, University of Oslo, Norway; 2 Department of Infectious Diseases, Oslo University Hospital, Norway; 3 Department of Infectious Disease Immunology, Statens Serum Institut, Copenhagen, Denmark; 4 K. G. Jebsen Center for Inflammation Research, University of Oslo, Norway

## Abstract

Tuberculosis (TB) has huge impact on human morbidity and mortality and biomarkers to support rapid TB diagnosis and ensure treatment initiation and cure are needed, especially in regions with high prevalence of multi-drug resistant TB. Soluble interferon gamma inducible protein 10 (IP-10) analyzed from dry plasma spots (DPS) has potential as an immunodiagnostic marker in TB infection. We analyzed IP-10 levels in plasma directly and extracted from DPS in parallel by ELISA from 34 clinically well characterized patients with TB disease before and throughout 24 weeks of effective anti-TB chemotherapy. We detected a significant decline of IP-10 levels in both plasma and DPS already after two weeks of therapy with good correlation between the tests. This was observed both in pulmonary and extrapulmonary TB. In conclusion, plasma IP-10 may serve as an early biomarker for anti-TB chemotherapy responses and the IP-10 DPS method has potential to be developed into a point-of care test for use in resource-limited settings. Further studies must be performed to validate the use of IP-10 DPS in TB high endemic countries.

Tuberculosis (TB) is a major global health problem. An estimated 9 million people develop TB disease and 1.5 million die of TB every year despite the fact that TB is a preventable disease^1^. The limitation of diagnostic tools and insufficient follow-up of treatment pose a major threat for the individual patient as well as to global health. There is an urgent need of biomarkers to predict disease progression from latent tuberculosis (LTBI), treatment efficacy and cure and as read-outs for immune responses to immunization^2^. Multi-drug resistant (MDR)-TB poses a threat to global control of TB^3^,^4^ and has reinforced the need to find biomarkers that indicate relapse-free treatment success^5^.

Interferon gamma inducible protein 10 (IP-10/CXCL-10), a pro-inflammatory chemokine involved in leucocyte migration and activation, is a promising candidate as surrogate biomarker in TB infection^6^. IP-10 has been investigated in a broad spectrum of infectious and non-infectious chronic inflammatory diseases^7^. The diagnostic accuracy of IP-10 for TB infection seems to be on par with interferon gamma (IFN-γ)-release assays (IGRAs), but plasma IP-10 is expressed at a much higher level than IFN-γ and thus considered a more robust marker^8^. Data from both animal^9^,^10^ and human studies^11^,^12^,^13^,^14^,^15^,^16^,^17^ suggest that plasma IP-10 also has potential as a biomarker for therapy responses in TB.

The World Health Organization (WHO) and Stop TB Partnership have assigned 2015 for developing a simple point-of-care (POC) test to ensure rapid TB diagnosis and treatment initiation. The potential impact, future applications as well as challenges of POC tests have recently been reviewed by Dheda *et al*^18^. The filter paper method, carried out either as dry blood spots (DBS) or dry plasma spots (DPS), has simplified diagnostics of several diseases in resource-constrained settings^19^,^20^. However, to our knowledge, there are no previous studies of IP-10 DPS during anti-TB chemotherapy. Thus, we aimed to compare the IP-10 DPS method with the direct measurement of IP-10 in plasma analyzed by ELISA and investigate whether the IP-10 DPS method could be used to monitor treatment efficacy in TB infection. We report for the first time that plasma IP-10, as measured by both methods, declined in response to anti-TB chemotherapy. Thus, our data indicate that the IP-10 DPS assay has potential as a biomarker for treatment responses and TB cure and may be developed into use as a POC test in resource-limited settings.

## Results

### Study participants

Thirty-four TB patients were longitudinally followed during 24 weeks of anti-TB chemotherapy. Thirty-two patients were *Mycobacterium tuberculosis* *(Mtb)* culture-confirmed whereas in two patients diagnosis was based on clinical suspicion, findings on X-ray and a positive QuantiFERON-TB test. Demographic and clinical characteristics at inclusion are summarized in Table 1. All patients were HIV-negative. There were 14 patients with pulmonary TB (PTB) and 14 with extrapulmonary TB (EPTB), 3 with combined PTB and EPTB, and 3 patients with disseminated TB. Sixteen patients were classified into the “low symptom score” group and 18 patients into the “high symptom score” group. There was a tendency of a higher symptom score in PTB compared to EPTB patients (p = 0.057), in keeping with a higher proportion of PTB patients with an erythrocyte sedimentation rate (ESR) >40 mm/hour (above the median ESR for the cohort) (77% vs. 23%, p = 0.017).

Thirty-one patients received standard first-line anti-TB chemotherapy (isoniazid, rifampicin, pyrazinamide and ethambutol) whereas two patients received modified therapy with rifampicin, pyrazinamide and ethambutol due to isoniazid resistance and one patient received isoniazid, rifampicin and ethambutol due to pyrazinamide resistance. Five patients received extended standard anti-TB chemotherapy for 9–12 months due to EPTB, disseminated TB disease, drug resistance or lack of sputum conversion after 2 months of therapy.

After 8 weeks of treatment, 13/14 (93%) of the PTB patients were *Mtb* culture negative, whilst all PTB patients were *Mtb* culture negative after 12 weeks. All patients demonstrated clinical improvement of TB disease during treatment, but four patients with glandular TB experienced transient paradoxical increase of the lymph nodes. In addition, two patients experienced worsening of gastrointestinal symptoms during anti-TB chemotherapy; one with known Crohn's disease and one diagnosed with Crohn's disease shortly after initiation of treatment. In both cases abdominal TB was excluded. For the entire group of patients the ESR levels declined significantly from baseline to the end of treatment [44 mm (5–111) to 8 mm (1–52), p < 0.001].

### Decline of plasma IP-10 during anti-TB chemotherapy

**Figure 1:**
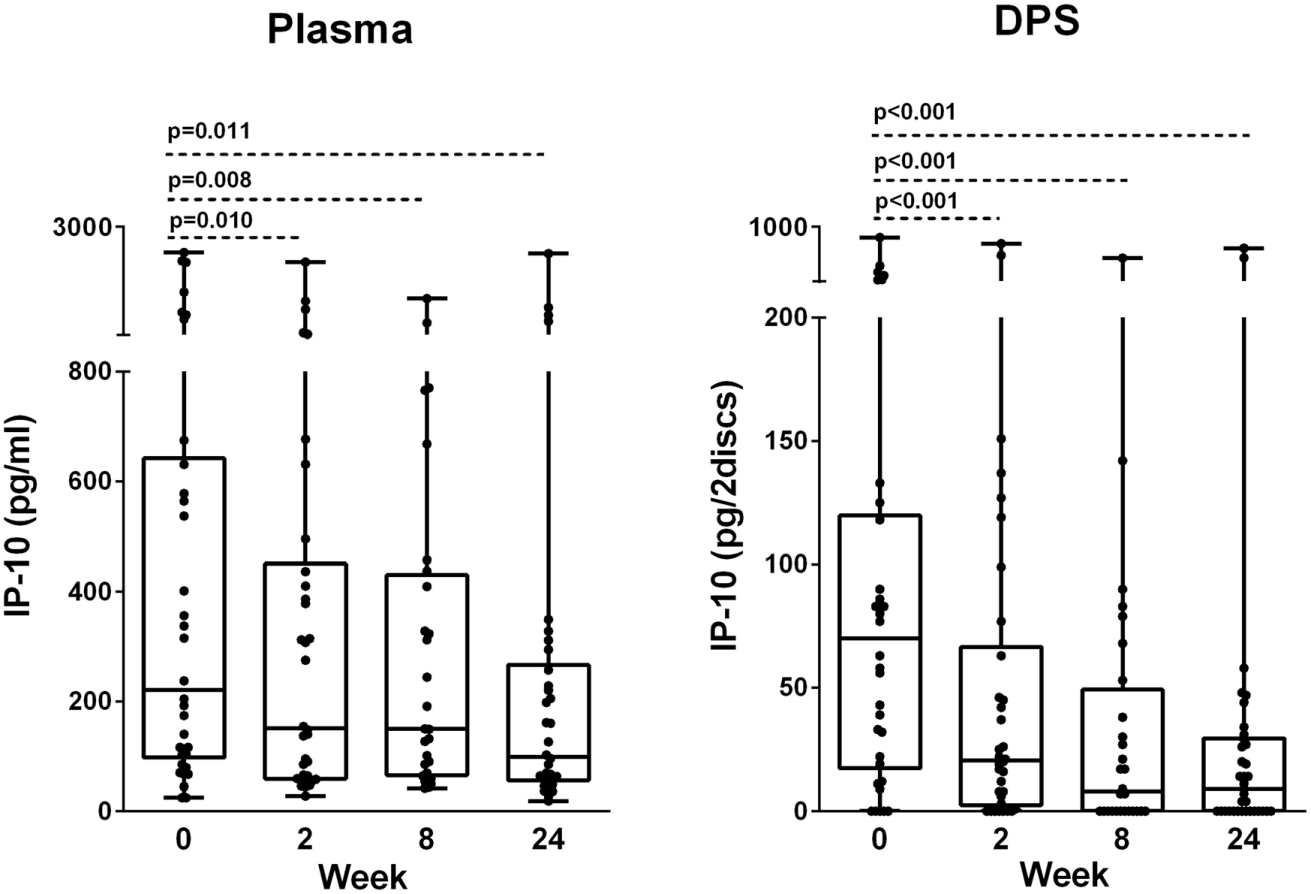
Dynamics of IP-10 in plasma and Dry Plasma Spots (DPS) during anti-TB chemotherapy. IP-10 levels measured in plasma and DPS in patients with active TB during 24 weeks of effective anti-TB chemotherapy: Baseline (week 0) (N = 34), week 2 (N = 34), week 8 (N = 28), week 24 (N = 34). Box-whisker plots with median, interquartile ranges and min/max values indicated. P-values were calculated by Wilcoxon Signed Ranks test. A significance level of 0.05 was used.

**Figure 2:**
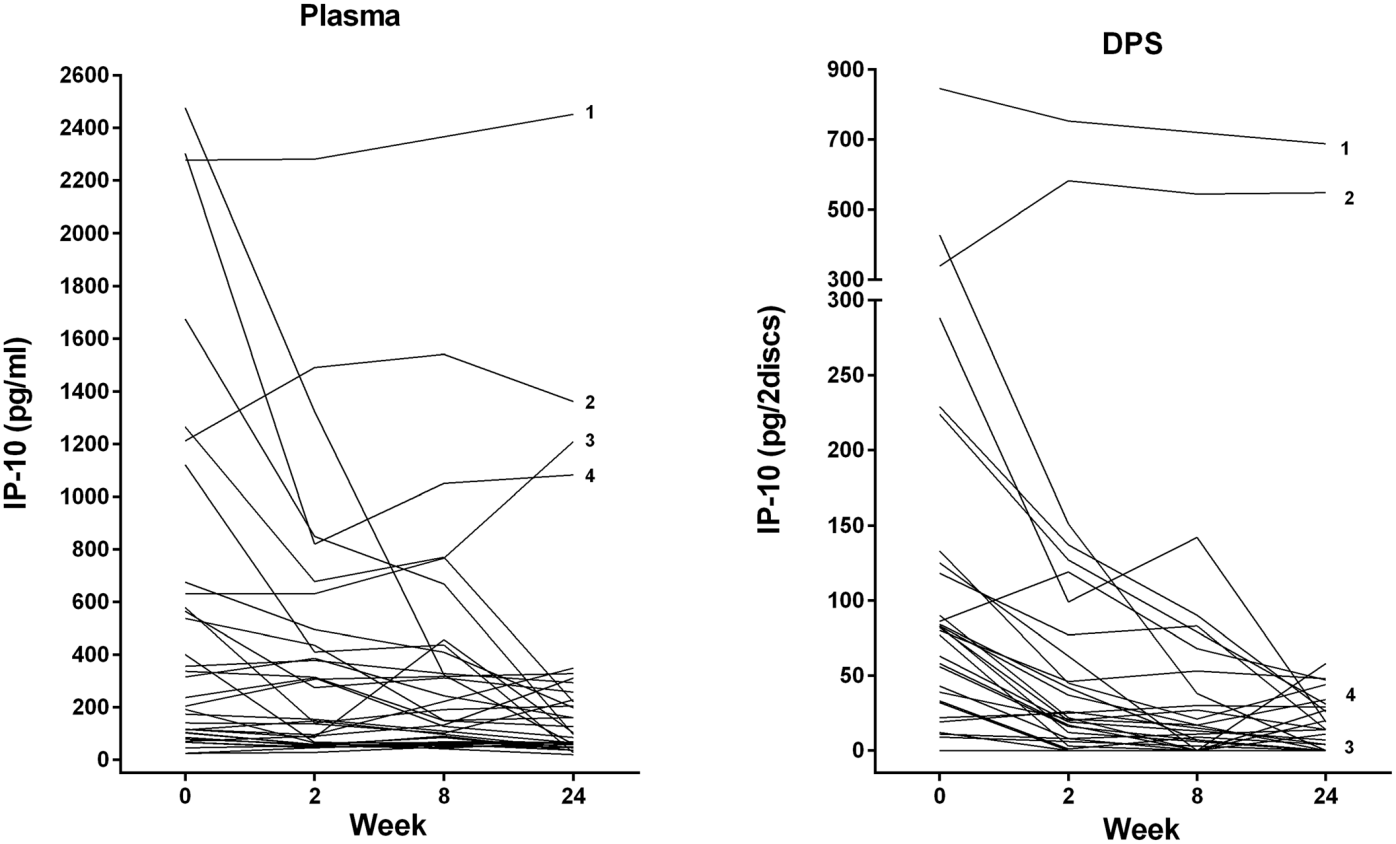
Comparison of Plasma and Dry Plasma Spots (DPS) IP-10 in Individual patients during anti-TB chemotherapy. IP-10 levels during 24 weeks of effective anti-TB chemotherapy in (A) plasma and (B) DPS in patients with active TB (N = 34): Baseline (week 0) (N = 34), week 2 (N = 34), week 8 (N = 28), week 24 (N = 34). ^1^Patient with pulmonary TB, previously treated (2 years earlier), but responding to present therapy with negative culture, ^2^Patient with Crohn's disease diagnosed during TB treatment, ^3^Patient with paradoxical reaction of glandular TB during treatment, ^4^Patient with disseminated TB and exacerbation of known Crohn's disease from week 2.

In the *plasma samples* we observed an overall significant reduction in IP-10 levels during 24 weeks of treatment (p = 0.004). Plasma IP-10 declined significantly already from baseline to week 2 [221 pg/ml (98–642) vs. 151 pg/ml (59–451), p = 0.010] and to week 24 [99 pg/ml (56–269), p = 0.011] (Fig. 1). Interestingly, IP-10 levels in the two patients with Crohn's disease as well as in one patient with PTB responding to therapy with negative culture, but who had also been treated for TB two years earlier, remained persistently elevated during treatment (Fig. 2A). An apparent increase in IP-10 levels were also seen in one of the EPTB patients who experienced paradoxical lymphadenopathy during therapy, whereas patients with disseminated and resistant TB responded with decline in IP-10 levels corresponding to clinical improvements.

### Decline of IP-10 from Dried Plasma Spots (DPS) during anti-TB chemotherapy

In the *dried plasma spots*, we also observed a corresponding and overall significant reduction in IP-10 levels during treatment (p < 0.001). DPS IP-10 levels declined significantly already at week 2 [70 pg/2discs (17–120) vs. 20 pg/2discs (3–67), p < 0.001], but in contrast to plasma IP-10, an additional decline was observed at week 8 [8 pg/2discs (0–49), p = 0.011] with stable levels towards week 24 [9 pg/2discs (0–30), p < 0.001] (Fig. 1). A corresponding pattern as seen for individual patients in the regular plasma IP-10 analyses was also found by the DPS IP-10 method, but only the previously treated TB patient and one of the patients with Crohn's disease maintained IP-10 at high levels (Fig. 2B).

### Plasma IP-10 and DPS IP-10 correlate during anti-TB chemotherapy

**Figure 3:**
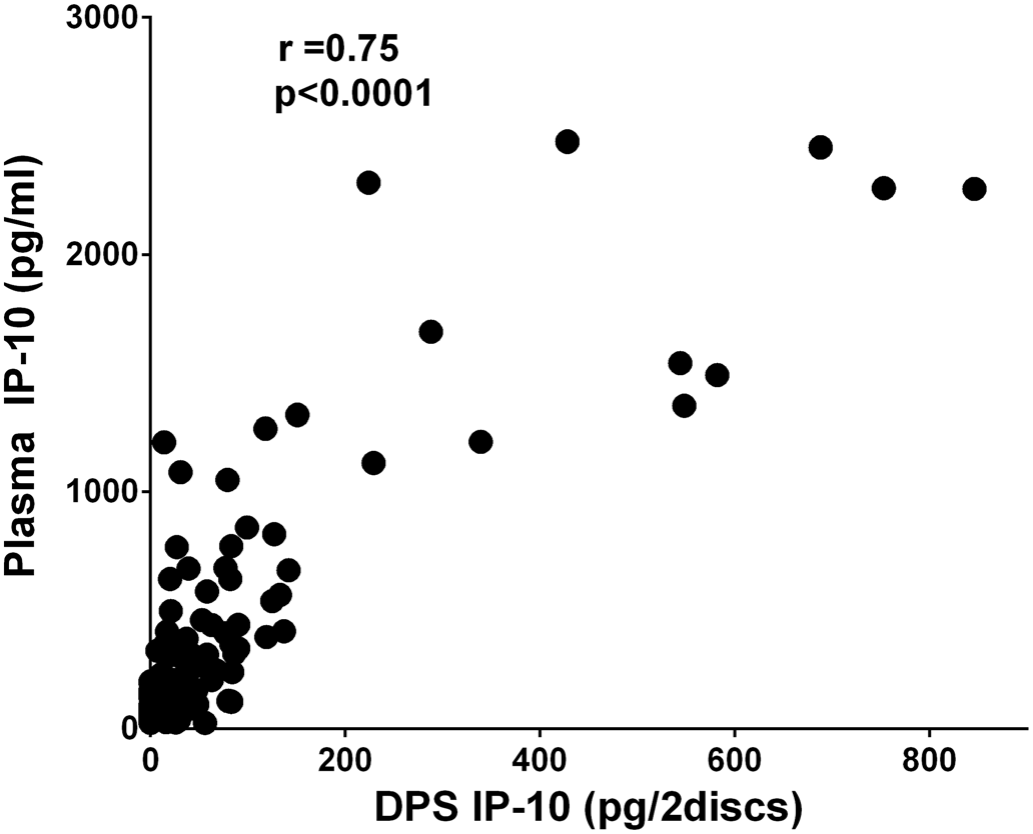
Correlation between IP-10 levels measured in plasma and Dry Plasma Spots (DPS). Correlation between plasma samples and DPS for all TB patients at all time-points during treatment (baseline, 2, 8 and 24 weeks). Correlation coefficients were calculated by Spearman's rho (significant values at the 0.01 level, 2-tailed).

IP-10 levels measured directly in plasma and after extraction from DPS correlated well throughout treatment (r = 0.75, p < 0.0001) (Fig. 3). The same positive correlation between IP-10 levels measured by the two methods was found in the PTB (r = 0.75, p < 0.001) and EPTB (r = 0.77, p < 0.001) subgroups.

### IP-10 levels according to symptom score and TB disease localization

**Figure 4:**
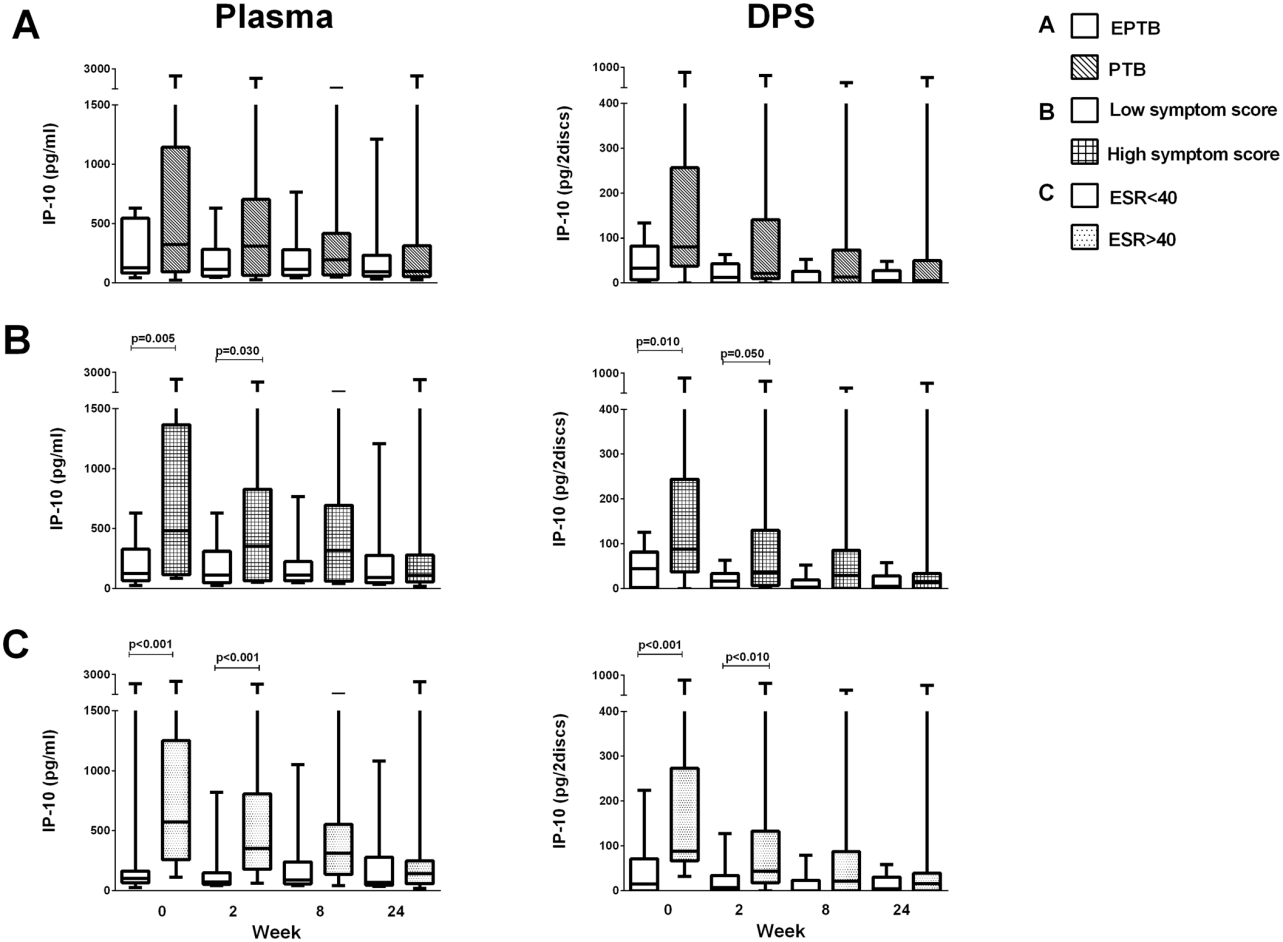
IP-10 levels in clinical TB subgroups during anti-TB chemotherapy. Plasma IP-10 and Dry Plasma Spots (DPS) IP-10 during 24 weeks of anti-TB chemotherapy in (A) extrapulmonary TB (EPTB, N = 14, white box) and pulmonary TB (PTB, N = 14, hatched box), (B) patients with “low symptom score” (N = 16, white box) and “high symptom score” (N = 18, hatched box) and (C) Erythrocyte Sedimentation Rate (ESR) < 40 (N = 16, white box) and >40 (N = 16, hatched box). Box-whisker plots with median, interquartile range and min/max values indicated. P-values were calculated by Mann-Whitney U test. A significance level of 0.05 was used.

Plasma IP-10 levels were analyzed in different clinical subgroups of the study cohort. At baseline, the PTB group had numerically, but not statistically, higher median IP-10 levels compared to the EPTB group both in plasma samples [326 pg/ml (94–1144) vs. 128 pg/ml (84–544), p = 0.29] and in DPS samples [80 pg/2discs (38–257) vs. 33 pg/2discs (7–83), p = 0.064] (Fig. 4A). Further, we found at baseline significantly higher levels of IP-10 in TB patients with “high symptom score” than in patients with “low symptom score” both in plasma [483 pg/ml (115–1367) vs. 128 pg/ml (67–326), p = 0.005] and in DPS [88 pg/2discs (37–244) vs. 45 pg/2discs (2–82), p = 0.010] (Fig. 4B). Finally, patients with ESR > 40 had also significantly higher IP-10 levels than patients with ESR < 40 both at baseline and after two 2 weeks (Fig. 4C).

## Discussion

Biomarkers for monitoring of TB treatment are needed, especially for use in patients with MDR-TB with long-lasting, insufficient and demanding regimens. We report for the first time an early and parallel decline in plasma IP-10 levels after 2 weeks of efficient anti-TB chemotherapy. The decline was accurately detectable in DPS with good correlation between the two methods. Our data suggests that the easy and manageable IP-10 DPS method could be used to monitor treatment responses in patients with TB and possibly serve as a POC test in resource-limited settings.

IP-10 has been studied in patients with various stages of TB infection in samples from peripheral blood^21^,^22^,^23^,^24^,^25^,^26^, pleural fluid^27^,^28^,^29^, bronchoalveolar lavage^30^ and urine^11^. IP-10 may also improve TB diagnosis both in HIV infection^31^,^32^,^33^ and in children^34^,^35^,^36^. However, IP-10 is not specific for TB disease^7^, but seems to be upregulated in many pro-inflammatory conditions including Hepatitis C infection (HCV) where IP-10 has been used to predict the prospect of spontaneous clearance of acute HCV infection and treatment success in chronically infected patients^37^,^38^.

The decline in IP-10 levels as early as 2 weeks is in agreement with experimental data from mouse models suggesting that IP-10 is a responsive and dynamic biomarker^9^,^10^. In humans, Azzuri *et al.*^12^ found a significant decrease of plasma IP-10 in PTB patients after 2 months of treatment. A recent report by Hong *et al*. also found significant reductions in unstimulated serum IP-10 after 6–9 months of treatment in a group with low-risk for relapse of TB disease^17^. It has also been reported that IP-10 secretion after *in vitro* stimulation with RD1 antigens decrease after 6 months of anti-TB chemotherapy^16^.

The vast majority of patients with TB disease live in resource-limited settings in low-income countries with restricted capacity to diagnose TB disease and monitor treatment effect^39^. Sputum culture conversion after 2 months of therapy may predict non-relapsing cure, but has low sensitivity and modest specificity for predicting failure and relapse of TB disease^40^. This emphasizes the need for low-cost and easy-to perform diagnostic tools serving as POC tests that can be handled in settings without electricity and cold chain logistics. The filter paper method with DPS/DBS has previously been used in resource-limited settings to diagnose and monitor both HIV and CMV disease^19^,^20^. Aabye *et al*. have introduced a novel ELISA based method for quantification of IP-10 extracted from DPS/DBS and report a very good correlation to IP-10 levels measured directly in plasma^41^. The use of IP-10 was also assessed in a TB validation cohort, concluding that IP-10 was readily detectable in both plasma and DPS with excellent correlation^42^. Finally, IP-10 is proven to be a solid and robust marker in lateral flow assays delivering quantitative results within short time in resource-poor settings^43^. Still, to our knowledge, we are the first to explore the usefulness of DPS/DBS as biomarker for therapy efficacy in TB. Our results revealed a good correlation between IP-10 measured in plasma and DPS throughout the treatment period. Thus, IP-10 measured by DPS has potential for use as a field friendly tool allowing for real time monitoring of treatment efficacy in resource-constrained settings such as DOTS clinics, allowing for letter based sample transport for centralized analysis.

We have studied the performance of IP-10 in various clinical presentations of TB. EPTB is a diagnostic challenge; often pauci-bacillary in nature and difficult to access for biopsies from TB affected organs, especially during follow-up. We found higher IP-10 levels in PTB patients than in the EPTB group due to presumed higher levels of bacterial load in patients with PTB. However, this difference did not reach statistical significance, which could be explained by the heterogeneity of TB disease within the patient groups. Still, both clinical groups responded to therapy with a decline in plasma IP-10. Further, there were also significantly higher levels of IP-10 in the patients with “high symptom score” and patients with high ESR in accordance with studies showing elevated levels of IP-10 with increasing severity of disease^44^,^45^.

Biomarkers must reliably predict TB cure and respond to reactivation or therapy failure. Although IP-10 levels declined in most patients corresponding to bacterial clearance demonstrated by negative culture during follow-up, we observed fluctuating, incomplete or unsuccessful reduction in IP-10 levels in some of the TB patients, although not so apparent by the DPS method. This was seen in two patients who experienced worsening due to inflammatory bowel disease, but also in a patient facing a paradoxical reaction with an intensification of glandular TB after initiation of anti-TB chemotherapy. Increase in IP-10 levels have also been reported in patients who relapsed after treatment^12^ and IP-10 identified patients with high risk of cavitary TB disease as well as therapy failure with positive sputum smear after 2 months of treatment^46^. Altogether, these data show that the pro-inflammatory chemokine IP-10 can respond with a dynamic pattern to any sort of inflammation underscoring the importance to clinically investigate further patients who experience a non-satisfactory decline in IP-10 due to either treatment failure, TB relapse or intercurrent co-morbidities.

There are limitations in our study. The rather low numbers of patients give reduced power in the statistical calculations and increase the risk for Type 2 statistical errors. As our data indicate, co-morbidities may also influence upon the elevated IP-10 levels measured in this TB cohort. However, our patients were closely monitored during treatment and worsening of symptoms and co-morbidity was registered and correlated with the dynamics of IP-10 as described for individual patients. In addition, longer observations and measurements of IP-10 after completion of TB therapy is needed to evaluate the potential of IP-10 in predicting relapse of TB disease.

In conclusion our data suggest that an early decline of plasma IP-10 levels may serve as a biomarker for anti-TB chemotherapy efficacy. Further, as DPS IP-10 correlates well with plasma samples, this manageable diagnostic tool has potential to assist in monitoring treatment responses in resource poor settings. Thus, further prospective studies must be performed to validate the use of IP-10 DPS or DBS detection by POC tests in TB high endemic areas.

## Methods

### Study participants

Thirty-four patients with TB were included in the study (Table 1). Patients were categorized into pulmonary TB (PTB), extrapulmonary TB (EPTB), combined PTB and EPTB, and in disseminated disease. The patients were grouped as “low symptom score” (asymptomatic or with one of the following symptoms; fever >38C°, weight loss, wasting, cough or night-sweat) or high symptom score (2 or more symptoms). Blood samples were drawn into EDTA tubes at baseline and after 2, 8 and 24 weeks of standard anti-TB drug combination chemotherapy. Plasma was harvested after centrifugation, snap-frozen and stored at – 80C until analysis.

### Ethical considerations

The study was approved by the Regional Ethics Committee and written informed consent was obtained from all participants. All experiments were performed in accordance with relevant guidelines and regulations and the plasma was stored in the departments approved biobank.

### Filter Paper Sample Preparation

Preparation of the DPS was done according to protocol as described by Drabe *et al*.^47^. Plasma samples were thawed and spotted in 2 × 25 ul volume on Whatman 903 filter paper (Whatman, USA) per sample and allowed to air dry for 3–4 hours at ambient temperature. Filter paper samples were stored in zip-lock plastic bags with a desiccant and sent by postal service from Oslo University Hospital, Norway, to Statens Serum Instititute, Denmark.

### Protocol for IP-10 Determination in plasma and DPS Samples

Detection of IP-10 in plasma and DPS samples was performed by ELISA according to protocols developed by Aabye *et al*.^41^ and Drabe *et al*.^47^ respectively. In brief, plasma samples were diluted five times in dilution buffer (2% BSA, 0.1% Tween-20 in PBS) with HRP-conjungated detection mAb and allowed to incubate 2 hours in a mAb coated ELISA plate. For DPS samples, two 6 mm filter paper discs were punched from the DPS (Harris Puncher) and stacked horizontally in the bottom of a dry filter bottom plate (EMD Millipore, USA). Following, 80 ul dilution buffer was added to each well of the filter bottom plate and the DPS discs were allowed to rehydrate for 1 hour. Afterwards the filter bottom plate was stacked on top of a coated IP-10 ELISA plate with 20 ul dilution buffer with HRP-conjungated detection mAb and centrifuged at 4000 RPM for 5 minutes to force the DPS disc elute into the ELISA plate. The ELISA plate was then incubated 1 hour at room temperature. After incubation, plasma and DPS ELISA plates were washed × 3 in PBS with 0.1% Tween-20 followed by addition of HRP substrate (TMB One, Trichem, DK) and allowed to develop for 30 minutes before the color reaction was stopped and absorbance was read at 450 nm subtracted air blank 630 nm. Plasma levels were corrected for dilutions, DPS measurements are presented as pg/2 DPS discs.

### Statistical analysis

Statistical analyzes were performed by SPSS statistics 22 (IBM). Non-parametrical statistical methods were applied. Friedman test were used for measuring overall differences at different time points during treatment. For group-wise comparison Mann-Whitney U test was applied and for dependent variables the two-tailed Wilcoxon matched pair test. Correlation analyses were performed using Spearman's rank correlation coefficient. 2 × 2 Fischer exact test was used in analysis of categorical variables. A significance level of 0.05 was used. All values are presented as median and interquartile range [IQR]. Graphical presentations were made using Prism V5.04 and V6 software (GraphPad, San Diego, USA).

## Author Contributions

Conceived and designed the experiments: K.T., M.R. and A.M.D.R. Recruited the patients and collected clinical data: K.T. Performed the experiments: K.T. and M.R. Analyzed the data: K.T., M.R. and A.M.D.R.. Contributed reagents/materials/analysis tools: K.T., M.R., A.M.D.R. and D.K. Drafted and reviewed the manuscript: K.T., M.R., D.K. and A.M.D.R.

## Figures and Tables

**Table 1:** Patient characteristics (N = 34)

Age (median years, range)	30 (20–91)
Female (%)	15 (44)
Origin (%)	
Africa	15 (44)
Asia	15 (44)
Europe	4 (12)
**Localisation (%)**	
Pulmonary	14 (41)
Extrapulmonary^a^	14 (41)
Combined Pulmonary and Extrapulmonary^b^	3 (9)
Disseminated TB	3 (9)
Drug sensitive *Mtb*:Mono-resistant *Mtb*^c^	31:3
IGRA assay^d^ (positive:negative:no data)	21:0:13
ESR^e^ (median mm/hour, range)	39 (5–111)
Low:High symptom score^f^	16:18

^a^Lymphnode, pericard, abdominal, cutaneous abscess, osteomyelitis.

^b^Culture growth from both pulmonary and extrapulmonary specimens.

^c^Resistance for one of the standard first-line drugs.

^d^QuantiFERON®-TB.

^e^Erythrocyte Sedimentation Rate.

^f^High = ≥2 of the following symptoms: fever (>38C°), weight loss, wasting, cough and night-sweat. Low = 1 symptom or asymptomatic/detected by screening.
